# Clinical Assessment of Biphasic Calcium Phosphate in Granules and Paste Forms in Human Maxillary Sinus Bone Augmentation: A Randomized, Split-Mouth Clinical Trial

**DOI:** 10.3390/ma16031059

**Published:** 2023-01-25

**Authors:** João Paulo Bonardi, Rodrigo dos Santos Pereira, Carlos F. Mourão, Bruno Coelho Mendes, Adam Lowenstein, Pietro Montemezzi, Flavio Giubilato, Roberta Okamoto, Eduardo Hochuli-Vieira

**Affiliations:** 1 Department of Oral & Maxillofacial Surgery, Aracatuba School of Dentistry, Sao Paulo State University, Sao Paulo 16066-840, Brazil; 2 Department of Oral & Maxillofacial Surgery, University of Grande Rio-UNIGRANRIO, Rio de Janeiro 25071-202, Brazil; 3 Department of Periodontology, Division of Dental Research Administration, Tufts University School of Dental Medicine, Boston, MA 02111, USA; 4 Department of Dentistry, San Raffaele Hospital, 20132 Milan, Italy; 5 Department of Human Anatomy, Aracatuba School of Dentistry, Sao Paulo State University, Sao Paulo 16066-840, Brazil; 6 Department of Diagnostic and Surgery, Araraquara School of Dentistry, Sao Paulo State University, Sao Paulo 16066-840, Brazil

**Keywords:** biomaterials, bone substitutes, sinus floor augmentation

## Abstract

The aim of the present study is to compare the biphasic calcium phosphate (BCP) using two different forms—(1) granules and (2) paste—in human maxillary sinus bone reconstruction as a split-mouth study using histomorphometric and immunolabeling for osteocalcin. Ten patients with bilateral maxillary posterior partial edentulism were selected in order to reconstruct bone height. They were divided into two groups: BCPG and BCP-P. After six months of bone healing, biopsies were harvested to assess the new bone formation and immunostaining for osteocalcin. The BCP g group had the following results: mean of bone formation in pristine bone 49.4 ± 21.6%, intermediate 49.4 ± 16.2%, and apical 55.3 ± 21.4%. The group BCP-P had a mean of 41.9 ± 17.3% in the pristine bone region, 37.5 ± 7.8% for intermediate, and 39.0 ± 13.5% for apical. The osteocalcin immunolabeling was high for both groups, demonstrating bone calcification. Thus, the two biomaterials present suitable results for the placement of dental implants.

## 1. Introduction

A maxillary sinus lift using bone substitutes is a surgical procedure to increase the bone height in the posterior maxillary bone allowing for the placement of dental implants [1]. The surgical technique most used in the procedure was described by Boyne and James using the maxillary lateral wall approach with the Schneiderian membrane elevation [2].

The autogenous bone graft is still considered the gold standard for reconstructing this region due to its osteoconductive, osteoinductive, and osteoprogenitor features [3,4,5]. Researchers presented an ideal bone substitute because there was morbidity at the second surgical site and a limited amount of bone in the maxilla [6,7,8,9]. Different biomaterials have been studied throughout the years as allografts, xenografts, and alloplastic materials with promising results [10,11,12,13,14,15].

Two bone substitutes have characteristics of bone structure: Hydroxyapatite (HA) and beta-tricalcium phosphate (β-TCP). HA is similar to the bone structure in terms of osteoconductive behavior, biocompatibility, and bioactivity. However, HA’s resorption is slow when grafted in a biological environment [6,10,16]. Another biomaterial is β-TCP, an alloplastic bone substitute that has fast resorption and osteoconductive features [10,17,18].

The association of HA and β-TCP creates biphasic calcium phosphate (BCP), a biocompatible biomaterial with osteoconductive features. These features allow for the maintenance of the graft volume and suitable results in bone reconstruction [10,19,20]. This bone graft can be manufactured as granules or injectable paste allowing for a better outflow of the biomaterial in the bone defect [19].

The aim of the present study is to compare the BCP in a proportion of 60% of HA and 40% of β-TCP using two different forms—(1) granules and (2) paste—in human maxillary sinus bone reconstruction as a split-mouth study

**Hypothesis** **(H0).** *There is no difference in bone healing in maxillary sinus reconstruction using BCP in granules compared to the paste.*

## 2. Materials and Methods

### 2.1. Ethical Statement

The present study was performed in the Araçatuba Dental School—UNESP and approved by Ethical Committee with number 91334718.8.0000.5420. The CONSORT statement’s RCT checklist was used in order to assess the quality [21]. The research was conducted according to the principles of Helsinki’s declaration.

### 2.2. Number of Samples

The number of subjects studied was determined by a power test performed (http://calculoamostral.bauru.usp.br/, accessed on 10 September 2019) applied with a beta of 20% and an alpha of 5%. The standard deviation used was 9.9 and the mean difference of 14.8 according to previous research conducted in a one-tail test [22]. The results showed a minimum of nine subjects with bilateral maxillary sinuses requiring to be grafted for the present research.

### 2.3. Inclusion and Exclusion Criteria

The subjects were excluded if presented with uncontrolled periodontal disease; uncontrolled systemic disease; maxillary sinus pathologies; as a smoker; with residual roots in the maxillary sinus; and irradiated on the head and neck region for cancer treatment. Subjects were included with present bilateral maxillary sinuses requiring grafting, and with residual maxillary sinus floor bone ≤4 mm, determined by a cone beam computed tomography. The evaluation of bone loss was performed by two different researchers (J.P.B. and B.C.M.) from the present group.

### 2.4. Randomization

The choice for which side of the maxillary sinus would be grafted was conducted by a research clinical assistant and randomized at the website http://www.random.org (accessed on 13 November 2019).

### 2.5. Determination of the Groups to Be Evaluated

Eleven subjects with bilateral posterior maxillary edentulism requiring bone graft for height reconstruction were selected after the inclusion and exclusion criteria. The formation of the groups was as follows:

Group 1: 11 maxillary sinuses grafted with BCP-granules (BCP-G).
Group 2: 11 maxillary sinuses grafted with BCP-paste (BCP-P).

### 2.6. Surgical Procedure

The surgical procedures were performed by the same surgeon under local anesthesia using Mepivacaine 2% with epinephrine 1:100,000, according to Boyne and James [2]. A trapezoidal incision on the alveolar bone crest (cable no. 3 and BD lamina no. 15) was performed. It was followed by a periosteal mucosal detachment with a Molt Descaler (Duflex^®^, São Paulo, SP, Brazil) to expose the alveolar bone crest, allowing an adequate view of the maxillary sinus’ crest and anterior wall. The bone window was created through corticotomy with a diamond bur No. 6 (Wilcos^®^, Rio De Janeiro, Brazil). Afterward, this sinus membrane was gently detached from the floor of the maxillary sinus using proper curettes to avoid membrane damage. After the sinus membrane bilateral detachment, we randomly implanted the bone graft embedded in a sterile saline solution. Before surgical wound closure, a resorbable collagen membrane (Geistlich Bio-Gide^®^, Princeton, NJ, USA) was placed to protect the grafted area and guide the tissue regeneration, followed by a silk suture 4-0 (Ethicon^®^, Johnson & Johnson, Brazil), performed through interrupted points. In order to reduce pain, subjects were prescribed Tylenol^®^ 500 mg every six hours for three days. In order to reduce the chance of infection, subjects were prescribed Amoxicillin with Clavulanic Acid 875 mg/125 mg every 12 h for 10 days.

### 2.7. Samples Harvesting

Bone biopsies were harvested after 6 months of bone healing using a 3.0 mm × 15 mm trephine drill under local anesthesia and performed by the same surgeon as well. The direction of the drill was the same as the direction for dental implant placement. All of the samples were stored in a formalin solution (10%) for two days, keeping the apical–coronal orientation.

### 2.8. Histomorphometric Analysis

The laboratory steps were performed according to Pereira et al. [12] with the samples colored with hematoxylin and eosin. A researcher with experience in the field evaluated the samples in a light microscope with the images captures with 12.5× magnification by a digital camera (JVC TK1270, Tokyo Japan). The three regions evaluated of each sample followed Pereira et al. [13]: (1) pristine bone region, (2) intermediate, and (3) apical.

### 2.9. Immunohistochemical Analysis

The immunohistochemical assessment was completed according to Bonardi et al. [10] using primary polyclonal goat anti-human antibodies targeting osteocalcin (Santa Cruz Biotechnology, Santa Cruz CA, USA—SC18319). For signal magnification, a biotinylated donkey anti-goat antibody coupled with adivin was added to diaminobenzene as a chromogenic substrate. All images were captured with a digital camera attached to a light microscope. The data analysis was performed by a single evaluator with expertise in the area (R.O.). The evaluator’s scores were as follows: “0” for the absence of staining and “1”, “2”, or “3” for low, moderate, and intense, respectively.

### 2.10. Statistical Analysis

The histomorphometric evaluation outcomes were analyzed by the Shapiro–wilk test in order to determine homoscedasticity. After the normal distribution result, the ANOVA test was performed in order to compare the groups followed by Tukey’s multiple comparison test. Kruskal–Wallis test was performed if the data presented an abnormal distribution. A priori *p*-value < 0.05 was considered statistically significant (GraphPad Prism 9, San Diego, CA, USA).

## 3. Results

**Figure 1:**
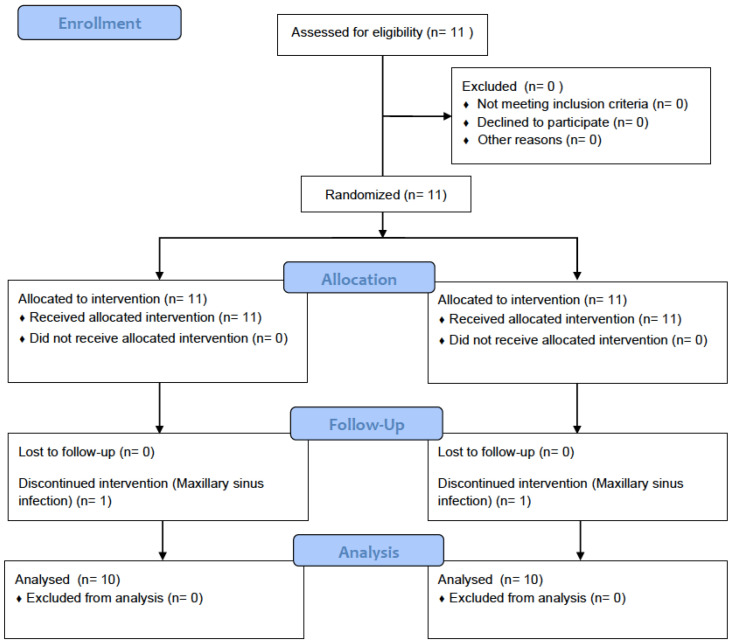
Study flow diagram.

Six females and five males (11) ranging from 52 to 64 years old underwent bilateral maxillary sinus bone height reconstruction using BCP-G and BCP-P in a split-mouth study. However, one patient had a post-surgical infection in the right maxillary sinus and was excluded from the research. (Figure 1).

### 3.1. Histomorphometric Results

**Figure 2:**
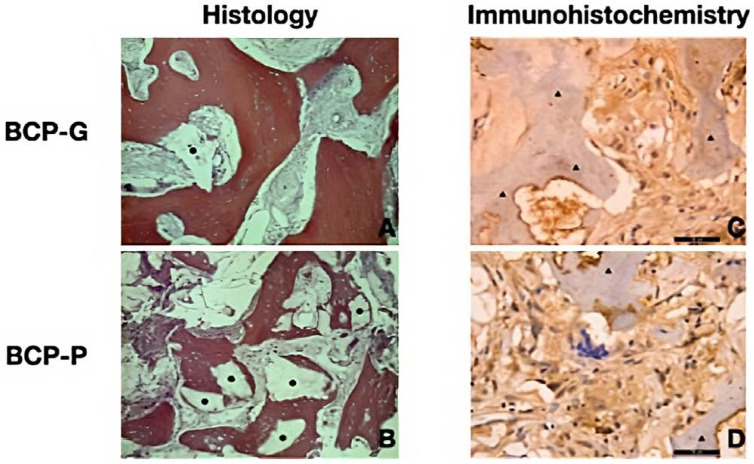
Image showing histological sections for BCP-G (**A**) and BCP-P (**B**). Immunolabeling for osteocalcin for BCP-G (**C**) and BCP-P (**D**). (**•**) Biomaterial remaining; (▲) Immunostaining for osteocalcin on bone surface. [Magnification, ×12.5 for **A**,**B**; ×40 for **C**,**D**].

**Figure 3:**
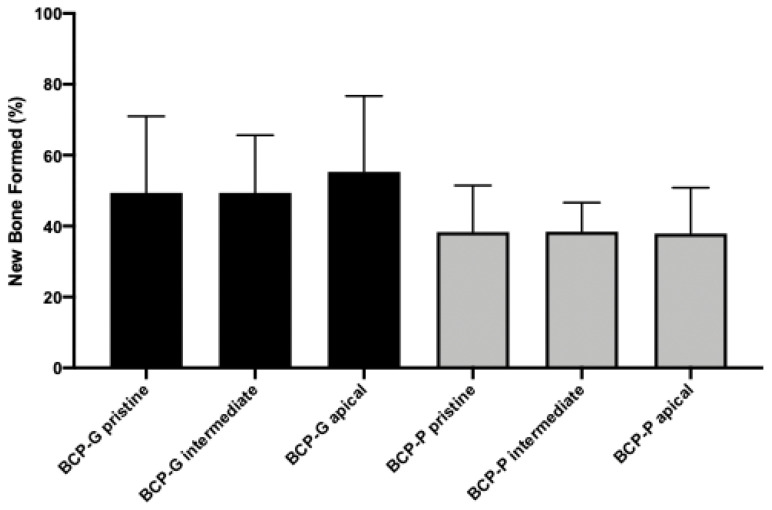
Graph showing the histomorphometric results of new bone formed in each region evaluated for BCP-G and BCP-P after six months of bone repair in human maxillary sinus augmentation.

**Figure 4:**
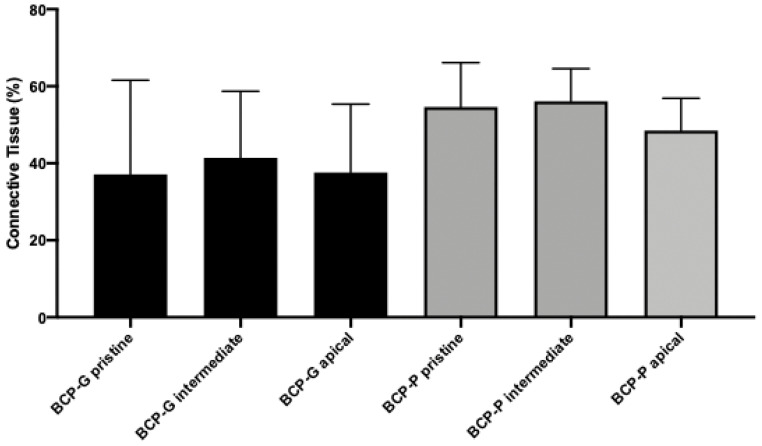
Graph showing the histomorphometric results of connective tissue in each region evaluated for BCP-G and BCP-P after six months of bone repair in human maxillary sinus augmentation.

The group BCP-G presented with a new lamellar bone formation and osteoblasts on the periphery and the areas of woven bone. The connective tissue presents as well cellularized, and few particles of the biomaterial could be observed (Figure 2A). The mean of new bone formed in the pristine bone area was 49.4 ± 21.6%. The intermediate and apical regions were 49.4 ± 16.2% and 55,3 ± 21.4%, respectively. The connective tissue presented with a mean of 37.1 ± 24.5%, 41.4 ± 17.3%, and 37.6 ± 17.7% in the pristine bone, intermediate, and apical regions, respectively. The median of biomaterial remaining was 3.5, 3.5, and 0 for pristine bone, intermediate, and apical regions, respectively (Table 1, Figure 3 and Figure 4). The BCP-P group presented similar histological findings as the BCP-G group with more areas of woven bone (Figure 2B). The mean of new bone formed was 41.9 ± 17.3% for the pristine bone region. The mean was 37.5 ± 7.8% for intermediate and 39.0 ± 13.5% for apical. The connective tissue mean was 51.0 ± 14.2%, 57.0 ± 8.7%, and 49.8 ± 7.3% for the pristine bone, intermediate, and apical regions, respectively. The median of biomaterial remaining for the present group was 2.5% in the pristine bone. The median was 0 and 1.5 for intermediate and apical, respectively (Table 2, Figure 3 and Figure 4). There is no statistical significance for the new bone formed (*p* = 0.07) and biomaterial remaining (*p* = 0.45). For the connective tissue formed, the ANOVA test showed statistical significance (*p* = 0.02). However, Tukey’s multiple comparison test could not find where the statistical significance occurred though.

### 3.2. Immunohistochemical Results

The immunostaining for osteocalcin was intense (score “3”) for both groups with yellow immunostaining on the bone surface. Therefore, the intense score “3” indicates bone maturation and calcification as well (Figure 2C,D).

## 4. Discussion

According to the handling of the biomaterials evaluated, the maxillary sinus grafting procedure was relatively easy using the BCP-P. However, when association with blood occurred, the paste became fluid and unstable, mainly related to the height required. Nevertheless, any post-surgical complication event was reported during the bone graft healing period.

When the HA was used for maxillary sinus bone graft, the outcomes demonstrated lower resorption as well as a lower mean of bone formation [13,23,24]. However, β-TCP has fast resorption with a suitable mean of bone formation compared with autogenous bone graft [11,21]. The BCP has the addition of HA and β-TCP, with the intention to combine the best properties of both biomaterials as well as a balance of bone formation and biomaterial resorption keeping the maintenance of the bone graft volume [25].

In the present study, it was possible to find the amount of biomaterial remaining. According to previous studies, the remaining biomaterial was higher using Bio-Oss when compared to both groups of the present research [26]. It is interesting because it is possible to have more new bone formation when the patient requires an implant; it will allow the specialist to have more bone contact for the implant surface. It is possible to find, in the literature, an essential role for implant survival when this one is placed in an area without biomaterials [27]. In contrast, a recent study did not show a significant relevance for implant survival or a higher prevalence of peri-implantitis in the grafted area. The study observed a similarity between the survival rate of implants used in the autogenous graft 97,9% (range: 95.6–100%) and 98,5% (range: 94.4–100%) in guided bone regeneration with non-autogenous bone substitutes [28]. However, bone-implant contact can help to distribute the occlusal stress in the maxillary molar areas, and it could avoid the risk of peri-implantitis [29]. Future studies should be carried out to understand better the correlation between implant survival and new bone formation in grafted areas using biomaterial with the potential for new bone formation, as demonstrated in the present research.

The use of BCP with 30% of HA and 70% of β-TCP in maxillary sinus lifting presented 34.93% of bone formation, 55.23% for connective tissue, and 9.82% of biomaterial remaining according to the literature [25]. The presence of connective tissue is a usual histological finding in areas of bone type IV, according to the Misch classification [1]. In addition, in those areas between connective tissue and new bone formation, osteoblasts can be observed in the bone matrix’s periphery in some cases [10,12,13,22,23]. These findings did not affect oral rehabilitation with dental implants in the present study or in other studies presented in the literature [10,12,13,22,23]. It is possible to observe similar data in other clinical studies. In an experimental study, Costa et al. performed a comparison between BCP in granules and paste in rabbit maxillary sinuses [23]. After 10 weeks, their results stated 34.20% for granules and 23.28% for paste, which differs from the present research outcomes with 49.4% and 41.9% for granules and paste, respectively.

Both biomaterials presented a suitable amount of bone formed with better results compared to the Ohayon study [27] for the granules group. Alternatively, a similar outcome for the paste was found in the present study in comparison to the study conducted by Olaecha et al. [25]. In the histological findings, it was possible to observe in Table 2 the absence of bone substitutes in both groups for some patients; this is due to the histology being a two-dimensional evaluation, and some slides did not show the remaining biomaterial.

Osteocalcin is a protein associated with bone maturation [25,30] and bone mineralization [30,31,32,33]. After six months, the bone healing was analyzed with osteocalcin, and there were suitable results for both groups indicating that are able to receive dental implants.

According to Helder et al. [34], the anatomy of the maxillary sinus changes from one patient to another. Thus, a split-mouth study was appropriate to compare these biomaterials. The limitations of the present research are as follows: (1) long-term evaluations of dental implants are required in order to demonstrate the clinical success of the biomaterials, and (2) the possibility of using immunostaining for osteoblast and vascular formation can be considered for a future study.

## 5. Conclusions

BCP in granules and paste form can be used for maxillary sinus elevation as a bone substitute. This is because of BCP’s suitable new bone formation and bone maturation after six months of bone healing, allowing for the placement of dental implants.

## Figures and Tables

**Table 1:** Histometric outcomes of BCP-G group after 6 months of bone repair.

Maxillary Sinus	New Bone Formed			Connective Tissue			Biomaterial Remaining		
	Pristine Bone (%)	Intermediate (%)	Apical (%)	Pristine Bone (%)	Intermediate (%)	Apical (%)	Pristine Bone	Intermediate	Apical
1D	47	27	60	22	39	40	25	36	0
2D	33	45	46	31	20	29	36	35	25
3D	87	49	73	13	49	27	0	2	0
4D	75	35	91	25	65	9	0	0	0
5E	73	62	23	20	35	50	7	3	27
6E	36	38	46	64	62	54	0	0	0
7E	52	67	63	6	29	27	42	4	0
8E	30	43	78	70	57	22	0	0	0
9E	33	81	33	48	14	67	19	5	0
10E	28	47	40	72	44	51	0	9	9
Mean	49.4	49.4	55.3	37.1	41.4	37.6			
SD	21.6	16.2	21.4	24.5	17.3	17.7			
Median							3.5	3.5	0

**Table 2:** Histometric outcomes of BCP-P group after 6 months of bone repair.

Maxillary Sinus	New Bone Formed			Connective Tissue			Biomaterial Remaining		
	Pristine Bone (%)	Intermediate (%)	Apical (%)	Pristine Bone (%)	Intermediate (%)	Apical (%)	Pristine Bone	Intermediate	Apical
1E	38	33	16	49	47	46	13	20	38
2E	23	23	20	45	42	51	32	35	29
3E	59	33	42	41	67	49	0	0	9
4E	33	42	41	55	58	59	12	0	0
5D	32	48	38	68	52	62	0	0	0
6D	37	39	44	63	61	53	0	0	3
7D	36	49	30	55	51	37	9	0	33
8D	24	32	58	71	68	42	5	0	0
9D	63	39	51	37	61	49	0	0	0
10D	74	37	50	26	63	50	0	0	0
Mean	41.9	37.5	39.0	51.0	57.0	49.8			
SD	17.3	7.8	13.5	14.2	8.7	7.3			
Median							2.5	0	1.5

## Data Availability

Not applicable.
